# High resolution, contrast-enhanced X-ray microscopy of the *ex vivo* human cochlea: technical feasibility and biometric case analysis

**DOI:** 10.3389/fnins.2026.1838204

**Published:** 2026-07-15

**Authors:** Sophie S. Jang, Charvi Malhotra, Stephen McInturff, Shelley Batts, Konstantina M. Stankovic

**Affiliations:** 1 Department of Otolaryngology-Head and Neck Surgery, Stanford University School of Medicine, Stanford, CA, United States; 2 Department of Neurosurgery, Stanford University School of Medicine, Stanford, CA, United States; 3 Wu Tsai Neurosciences Institute, Stanford University, Stanford, CA, United States

**Keywords:** cochlear microanatomy, contrast-enhanced imaging, *ex vivo* imaging, high-resolution imaging, otopathology, temporal bone, X-ray computed tomography (XCT), X-ray microscopy (XRM)

## Abstract

**Background:**

The human cochlea is a fragile, spiral-shaped sensory organ for hearing that is deeply embedded within the dense otic capsule bone, making accurate post-mortem anatomical studies difficult when using conventional histological approaches requiring sectioning. X-ray microscopy (XRM) is a non-destructive radiological imaging modality that combines geometric and optical magnification to achieve high-resolution 3D visualization.

**Methods:**

Here, we evaluated the ability of XRM to resolve the entire inner ear, including delineating soft tissue and bony structures, in trimmed human temporal bones *ex vivo* following lumen perfusion with a reversible iodine-based contrast-enhancing solution. After optimizing contrast incubation conditions, XRM datasets were acquired, reconstructed, and used to generate 3D volume renderings of the inner ear.

**Results:**

XRM enabled visualization of the entire cochlea and vestibular system, from millimeter-scale labyrinthine anatomy to submillimeter and micrometer-scale cochlear structures, including spiral ganglion nerve fiber bundles and sensory cell rows. Quantitative analysis in a representative case demonstrated an increasing width-to-height ratio of the organ of Corti from the basal to the apical cochlear turns, consistent with histology images and prior anatomical studies.

**Conclusion:**

These findings demonstrate that XRM provides high-resolution, sectioning-free *ex vivo* 3D visualization of human cochlear anatomy and may enable quicker and more faithful correlation of cochlear pathology with otologic disorders than traditional histology.

## 1 Introduction

Hearing loss is among the most prevalent sensory impairments, disabling roughly 5% of the world’s population or nearly half a billion people (World Health Organization, 2024). A challenge for the advancement of treatments and rehabilitative devices for hearing loss is the difficulty of accessing and analyzing the complex yet fragile human inner ear, which lies deeply embedded in the dense otic capsule within the temporal bone of the skull (Batts et al., 2025). Clinical computed tomography (CT) and magnetic resonance imaging (MRI) are indispensable for diagnosing many temporal bone and inner-ear disorders in patients, but their spatial resolution and soft-tissue contrast are insufficient to resolve most cochlear microanatomy, which appears as poorly differentiated soft tissue within the dense otic capsule (Batts et al., 2025; Leng et al., 2015; Yamashita et al., 2018). Consequently, detailed study of human inner ear structures (the cochlea and vestibular end-organs) has historically required post-mortem otopathology using embedded and physically sectioned temporal bones, where these delicate sensory structures reside, combined with direct cellular stains [e.g., hematoxylin and eosin (H&E)] (Tucci and Doherty, 2019).

Conventional otopathology has been highly valuable for understanding human cochlear anatomy in two dimensions (2D), as well as specific pathologies related to soft tissue (e.g., loss of sensory cells) and bony defects (Merchant et al., 2008; Tucci and Doherty, 2019). This approach has also helped to elucidate the anatomical consequences of the diverse etiologies of hearing loss related to aging, noise exposure, genetic mutations, infections, trauma, and other insults to the inner ear (Nadol, 1997; Sagers et al., 2017; Su-Velez et al., 2020; Tucci and Doherty, 2019; Viana et al., 2015). Although sectioning plus histology remains the “gold standard” for examining cellular morphology, it is time-intensive, risks introducing processing artifacts, and complicates the analysis of the complex 3D anatomy of the cochlea. Notably, although approximately 500 axial sections (20 μm in thickness) are required to span the entire human cochlea, only 5–7 reflecting the “golden view” of the mid-modiolus are typically examined (Mass Eye and Ear, 2026).

**FIGURE 1:**
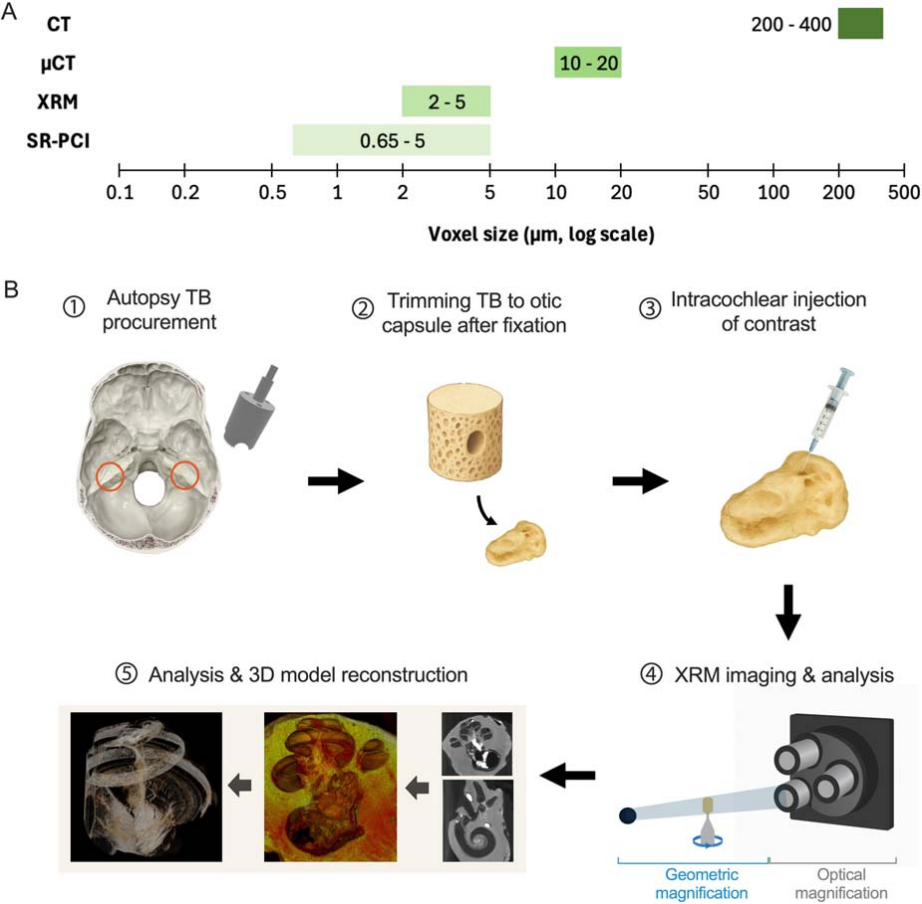
Relative resolution of radiological imaging modalities and X-ray microscopy (XRM) methodology. **(A)** The standard resolution ranges of the imaging methods examined in this study are plotted from lowest to highest resolution: clinical-grade computed tomography (CT), small animal research-grade micro-CT (μCT), XRM, and synchrotron radiation phase-contrast imaging (SR-PCI). Structural resolvability of cells is dependent on the size of the target area and the sampling voxel size. **(B)** Schematic of the experimental approach for XRM imaging of human temporal bone specimens, beginning with specimen procurement at autopsy. From the cylindrical temporal bone (TB) plug, the specimen was trimmed to the level of the otic capsule, leaving the inner ear fully encapsulated by the bone. Decalcification of bone was not required. Iodine, serving as a contrast agent, was perfused intracochlearly through the round window prior to XRM imaging. The resulting 2D projections were reconstructed in 3D to first visualize both soft tissue and bone, then further refined to capture the cochlea’s soft tissue complex anatomy. The icons of the bone plug and trimmed temporal bone were created using ChatGPT.

To bridge the gap between clinical imaging and sectioning-based otopathology, research-grade *ex vivo* imaging methodologies capable of penetrating dense bone have been applied to human temporal bone specimens, including CT and micro-CT (μCT) (Bommakanti et al., 2022; Khurayzi et al., 2021; Tang et al., 2018), synchrotron radiation phase-contrast imaging (SR-PCI) (Elfarnawany et al., 2017; Iyer et al., 2018; Lareida et al., 2009; Li et al., 2021; Rohani et al., 2020), and ultra-high-field MRI (Silver et al., 2002; Thylur et al., 2017). These volumetric approaches share the advantage of preserving the “intact” prepared specimen without physical sectioning, but differ substantially in spatial resolution, field of view (FOV), soft-tissue contrast mechanisms, contrast-agent requirements, accessibility, and costs (Batts et al., 2025). They have been used for tonotopic mapping of the cochlea and gross 3D cochlear anatomical modeling (Sagi et al., 2023; Tucci and Doherty, 2019); for more details, refer to our comprehensive review of clinical and research-grade imaging methods for the human inner ear (Batts et al., 2025). Among the methods, only SR-PCI has demonstrated cellular-level resolution rivaling that of conventional histology in human specimens (Figure 1A; Iyer et al., 2018; Lareida et al., 2009), but it requires access to large and costly synchrotron infrastructure (Withers et al., 2021). While clinical CT and μCT are more accessible to researchers, they provide lower soft-tissue contrast or resolution for delicate cochlear structures.

X-ray microscopy (XRM), also referred to as X-ray computed tomography (X-ray CT or XCT) in interdisciplinary contexts, is a laboratory-based imaging modality that combines geometric and optical magnification to generate high-resolution 3D images of internal structures (Withers et al., 2021). Among research-grade inner ear imaging approaches, XRM occupies an intermediate position between conventional CT/μCT and synchrotron-based imaging, offering volumetric visualization of optically opaque or mineralized *ex vivo* specimens without physical sectioning. This sectioning-free approach preserves the 3D architecture and spatial relationships of fixed tissue preparations while providing greater accessibility than synchrotron-based methods (Mayo et al., 2003). However, its distinguishing feature is the combination of geometric and optical magnification, which can achieve micron- to sub-micron voxel sizes via additional optical magnification across specimen FOV similar in size to the human cochlea (Cnudde et al., 2008; Sakdinawat and Attwood, 2010; Stock, 1999), while requiring substantially lower X-ray doses than synchrotron-based imaging like SR-PCI (Withers et al., 2021). XRM is more readily accessible than synchrotron-based systems due to its much smaller size (1.5 m vs. 1–27 km for synchrotron) and lower cost of the device (e.g., < $1 million for Zeiss Xradia Versa 520 vs. > $500 million for synchrotron) (Withers et al., 2021). Unlike SR-PCI, which can exploit phase-contrast effects to visualize soft tissues without exogenous staining, laboratory-based XRM typically requires contrast enhancement to distinguish delicate cochlear soft tissues from surrounding fluid and bone. However, reversible iodine-based contrast enhancement (Bommakanti et al., 2022) can preserve the prepared specimen for selected downstream analyses after XRM imaging (i.e., is “non-destructive” to the specimen). Thus, the rationale for evaluating contrast-enhanced XRM for otopathology is that, in addition to avoiding histological sectioning, it may also provide a more accessible laboratory-based platform for high-resolution 3D visualization of fixed human cochlear specimens.

Here, we evaluated contrast-enhanced XRM as a sectioning-free *ex vivo* approach for visualizing human cochlear microanatomy within fixed postmortem temporal bone specimens. Using reversible Lugol’s solution to enhance soft tissue contrast, we acquired high-resolution 3D datasets of trimmed temporal bone specimens with the inner ear (cochlea and vestibular system) retained *ex vivo*. We then assessed whether XRM-derived images could support quantitative morphologic measurements through a proof-of-concept biometric case analysis of the human organ of Corti.

## 2 Materials and methods

### 2.1 Human temporal bone procurement and processing

Human temporal bones were procured through the Research Autopsy Center at Stanford University and processed as previously described (Sagi et al., 2023) and as depicted in Figure 1B. Briefly, the calvarium was removed and the brain was lifted from the cranial vault. A circular autopsy saw was used to extract the temporal bone plug, which is ∼1.5 inches in diameter, using the internal auditory canal and arcuate eminence as anatomical landmarks. The temporal bone plug was immediately placed in 4% paraformaldehyde and stored at 4°C for 14 days. Decalcification of the temporal bone was intentionally omitted. Following fixation, an otologic surgical hand drill (eMax2, Anspach, FL, United States) was used to trim the bone plug to the level of the otic capsule, leaving minimal bone remaining while preserving the inner ear’s soft tissue architecture. Trimming the temporal bone plug reduces background X-ray attenuation by removing excess highly-absorbing obstacles like calcium in bone, thereby improving contrast enhancement of cochlear soft tissues.

### 2.2 Contrast enhanced XRM

#### 2.2.1 Lugol’s stain preparation and incubation

Stock solution of Lugol’s stain (I_2_KI, 0.5% I_2_ and 1% KI) was prepared as previously described (Bommakanti et al., 2022). Briefly, KI was dissolved in distilled (DI) water overnight and pulverized I_2_ was added to the solution. After adding the remaining DI water volume, the solution was stirred until fully dissolved and then passed through a qualitative filter paper. Stock solution was stored at room temperature and protected from light.

The fixed, trimmed temporal bones were gently perfused with the prepared Lugol’s stain solution through the round window using a 28G syringe then submerged in the Lugol’s stain solution. To identify the optimal incubation time in the stain solution, incubation periods of 72, 96, 120, 144, and 216 h were evaluated. The optimal incubation time was determined by qualitative assessment of 2D and 3D reconstructed images, including well-defined visualization of soft tissue enhancement (e.g., spiral ganglion nerve fiber bundles and the organ of Corti) and minimal non-specific enhancement of bone.

#### 2.2.2 XRM imaging parameters and 3D image reconstruction

XRM of the trimmed human temporal bones was performed using a Zeiss Xradia 510 Versa system (Zeiss, CA, United States) with 0.4x and 4x magnification objectives. For imaging at 0.4x magnification, the specimens were placed at a median of 83.2 (interquartile range [IQR]: 108.6) mm from the detector and 71.5 (38.7) mm from the X-ray tube, also known as the source. A median of 100.1 (IQR: 20) kV of X-ray was delivered with an LE6 filter, which is equivalent to a 1.3 mm aluminum filter. Mode of 1600 projections was collected over 360°. Median exposure time per projection was 1 s (IQR: 4.0) and the total scan duration was approximately 30–60 min. For imaging at 4x magnification, the specimens were placed at a median of 38.9 mm (IQR: 12.7) mm from the detector and 179.5 (85.0) mm from the source. A median of 138.9 (IQR: 15.0) kV of X-ray was delivered with HE1 and HE2 filters, which are high-energy filters to reduce beam-hardening artifacts. Mode of 2400 projections was collected over 360°. Median exposure time per projection was 20.0 s (IQR: 11.3) and the total scan duration ranged from approximately 8.9–13.3 h depending on the FOV and optical magnification. Radiation exposure was considered in relation to preservation of fixed specimen integrity and compatibility with downstream analyses, not in relation to living tissue tolerance.

Reconstruction was performed using the Zeiss Reconstructor Scout-and-Scan Control System software (v16.2.18058.47373, Zeiss, CA, United States), as well as ImageJ software (v1.53k, National Institute of Health, MD, United States) with the Fiji XRM Reader plugin (v1, Vienna, Austria) (Metscher, 2022).

### 2.3 3 D model reconstruction and biometric analysis

#### 2.3.1 XRM 2D image analysis and 3D modeling

Reconstructed XRM imaging sequences were imported into Osirix (v14.1.1, Geneva, Switzerland) for further analysis. All quantitative analysis was performed using a 2-axis mid-modiolus view to achieve an orthogonal view using the Multiplanar Reconstruction tool. The 3D Volume Rendering tool was used for 3D modeling. Raw grayscale tomograms were mapped to color look-up table set to 16-bit. Parameters were adjusted to optimize the visualization of gross and microanatomical structures.

#### 2.3.2 Comparison of XRM with other radiological imaging methods

To perform qualitative comparative radiological studies, image sequences using three other imaging modalities (clinical CT, μCT, and SR-PCI) were obtained and 3D modeling performed as described above for XRM imaging. Specifically, clinical-grade CT series of temporal bone internal auditory canal were obtained, deidentified, and analyzed. μCT images of a temporal bone plug procured during an autopsy described in the ‘Human temporal bone procurement and processing’ section were also deidentified and analyzed. SR-PCI data of a human cochlea were obtained from Iyer et al. (2018) and analyzed.

#### 2.3.3 Biometric analysis of the organ of Corti using XRM in a representative case study

Biometric analysis, including height and width measurements and ratios, were performed on fully intact human organ of Corti using XRM and H&E-stained sections. XRM 2D orthogonal mid-modiolus views and celloidin-embedded histological mid-modiolus sections of the cochlea, with one specimen per modality, were used. Histology sections with H&E staining, the gold standard in otopathology, were previously shared by the National Temporal Bone Registry for a comparative study by Iyer et al. (2018). The specimens were matched on age, sex, and post-mortem interval (PMI) in each comparison.

Areas of the organ of Corti within fully intact lumens at the basal lower, basal upper, middle lower, middle upper, and apical turns of the cochlea were measured with ImageJ using the Measurement Tool. The width of the organ of Corti was defined by the distance medially from the inner hair cells and laterally to Hensen’s cells. Height was determined by the maximum perpendicular height extending to and inclusive of the basement membrane. The slope represents the rate of change of the organ of Corti’s width-to-height ratio as a function of the characteristic frequency position along the cochlear spiral from base to apex (as defined by the Greenwood function (Greenwood, 1990)). Although XRM enabled visualization of cochlear microanatomy, cellular features were less distinctly delineated than in H&E-stained sections; therefore, anatomical landmarks were used to guide structural identification. Each organ of Corti was measured in triplicate and the medians with IQRs were calculated.

### 2.4 Ethical approval

All research described in this article was conducted in accordance with the Declaration of Helsinki. Post-mortem human specimens were collected through the Research Autopsy Center at Stanford. Written informed consent for the use of tissue for scientific research was obtained from donors prior to death or from the legal next-of-kin by the Center. This research was determined to be exempt from institutional board review (IRB) as all specimens were derived from deceased individuals and fully deidentified.

Clinical CT research was approved by the Stanford University IRB-61 on 6/10/2024 (protocol #: 75803). The requirement for informed consent was waived due to retrospective nature of the study and the use of de-identified data, ensuring participant confidentiality.

## 3 Results

### 3.1 Human inner ear specimen procurement and optimization of Lugol’s stain incubation

Three human temporal bones from two individuals (Supplementary Table 1) were procured, processed with fixation and contrast, and underwent radiological XRM imaging (Figure 1B). The optimal incubation times of the specimens in contrast reagent (i.e., resulting in the best visualization of the cochlea’s soft tissue) were qualitatively determined to be 48 and 72–96 h for 0.4x and 4x magnification, respectively. Shorter incubations did not allow for sufficient penetration into soft tissue, while the degree of soft tissue enhancement plateaued at longer incubations. The incubation time needed for 4x imaging was dependent on the thickness of the bony otic capsule surrounding the cochlea, which affects X-ray penetration and detection (e.g., thicker bone results in longer incubation). The median voxel sizes were 24.5 (IQR: 23.6) μm and 5.2 (1.1) μm for 0.4x and 4x magnification, respectively.

### 3.2 Visual comparison of cochlear anatomy resolution between XRM and CT

**FIGURE 2:**
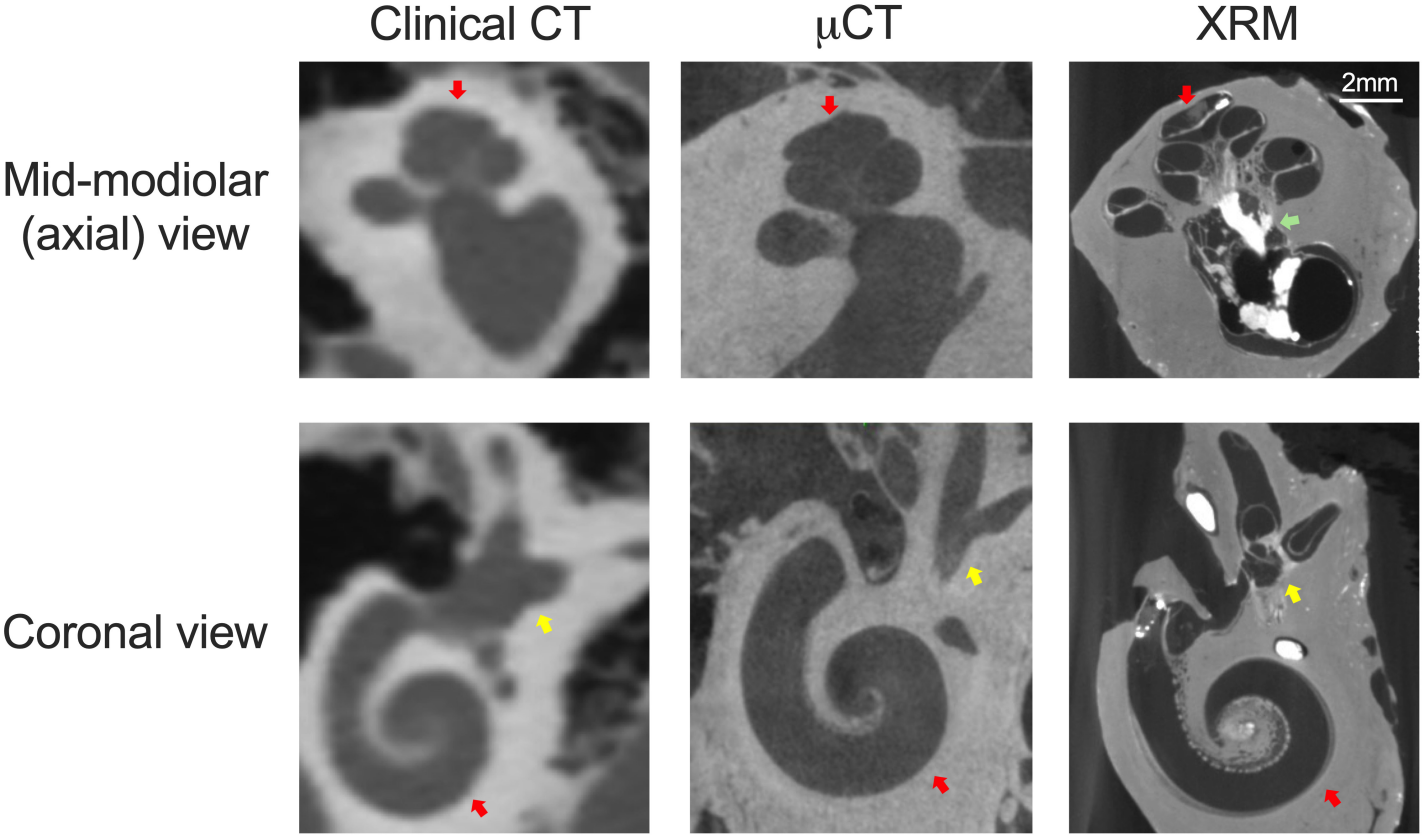
Comparison of the mid-modiolar (axial) and coronal views of the 3D intact cochlea using imaging modalities with increasing resolution. Specimens did not undergo decalcification which, due to the density of the human temporal bone and otic capsule, can require months. From left to right: clinical-grade computed tomography (CT), micro-CT (μCT), and contrast-enhanced X-ray microscopy (XRM), also known as X-ray CT. Scale bar is 2 mm for all panels. Arrow annotations are cochlea (red), vestibular system (yellow), and cochlear nerve (green).

We visually compared the ability of XRM with contrast to resolve the microanatomical structures of the human cochlea with that of standard clinical CT (the most common imaging modality for otologic investigations in patients) and μCT without contrast. The axial (mid-modiolar) and coronal 2D views of each modality are displayed in order of increasing resolution in Figure 2. Visual inspection showed that XRM produced superior delineation of soft tissue structures compared to the other modalities, including more refined gross anatomy and demarcated basilar membrane, Reissner’s membrane, spiral ligament organ of Corti and membranous labyrinth of the vestibular end-organs.

### 3.3 3 D reconstruction and modeling of the intact human inner ear using XRM

**FIGURE 3:**
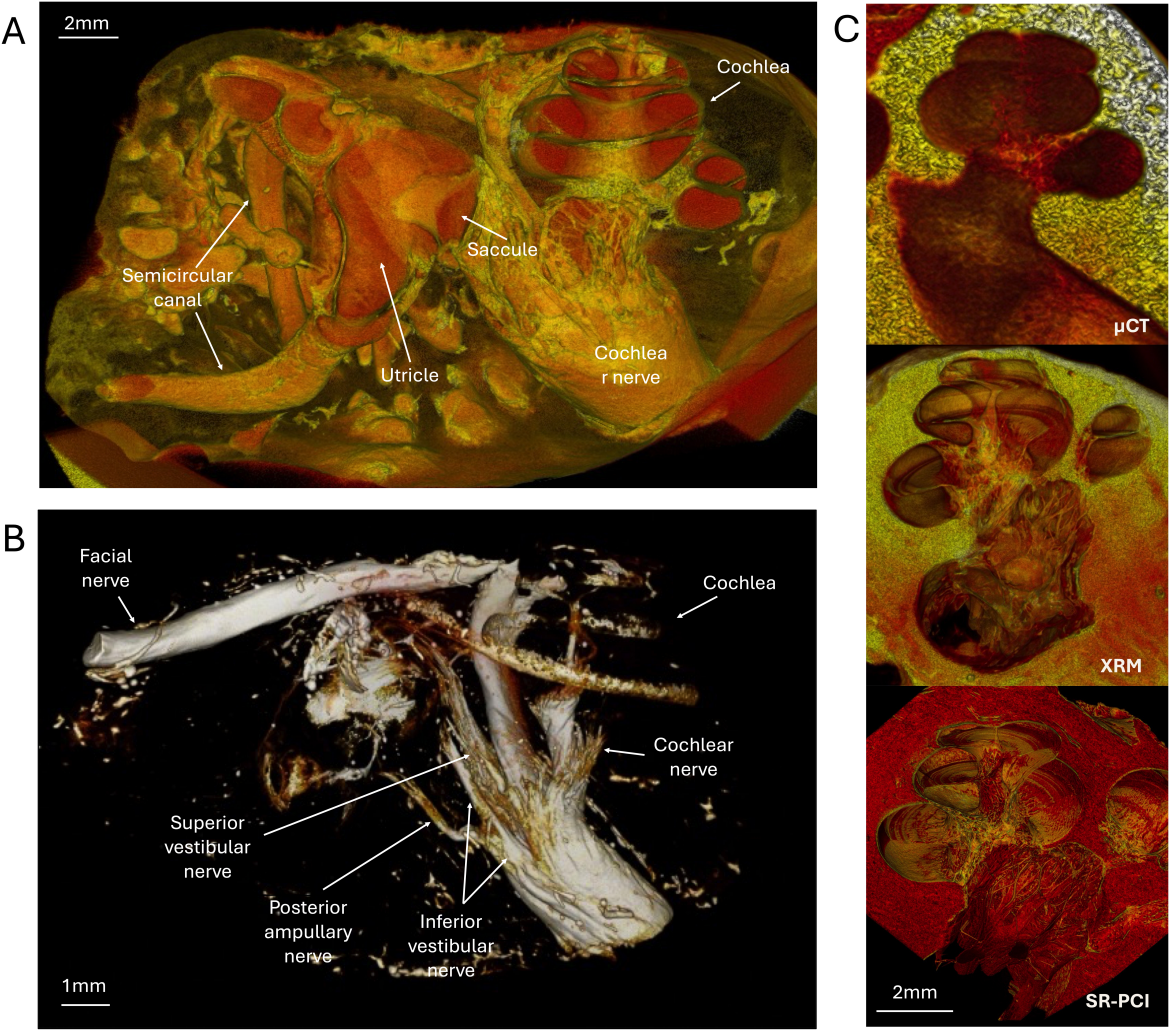
3D reconstructed XRM images of intact human temporal bone without decalcification at 0.4x magnification reveals the microanatomy of the cochlear and vestibular membranous labyrinth. **(A)** A labyrinthine view of the human inner ear with 3D XRM visualizes the cochlea, saccule, utricle, and a semicircular canal. **(B)** 3D XRM image reconstruction of the contrast-enhanced structures reveals individual branches of the vestibulocochlear nerve (i.e., cochlear, superior, and inferior vestibular nerves) and the facial nerve within the internal auditory canal. **(C)** Qualitative comparison of the resolution afforded from top to bottom by micro-computed tomography (μCT), XRM, and SR-PCI, demonstrating progressive improvement in definition between soft tissue structures and bone, the lumens of the cochlea, and cochlear substructures such as nerve fiber bundles and rows of inner and outer hair cells. **(A,C)** Pseudo-colored volume renderings generated from grayscale attenuation data to improve visualization of anatomical boundaries; color does not encode tissue type. Scale bar is 2 mm for all panels.

A cross-section of the cochlea, vestibule, and semicircular canals captured with XRM at 0.4x magnification revealed well-defined internal anatomical structures within the bony capsule, including nerve fibers in the mid-modiolus, saccule, and utricle (Figure 3A). When selecting for the contrast-enhanced soft tissues structures, grossly intact vestibulocochlear nerve and its branches (cochlear, superior vestibular, inferior vestibular with posterior ampullary nerve) and facial nerve were visualized (Figure 3B, 360° view in Supplementary Video 1). Research-grade radiological imaging modalities of the mid-modiolus cutout were compared across μCT with contrast, XRM with contrast, and SR-PCI (ordered from lowest to highest resolution) (Figure 3C). While gross anatomy was identifiable with μCT, images captured using XRM and SR-PCI showed greater delineation of the luminal compartments, nerve bundles as well as individual nerve fiber bundles, and the organ of Corti neatly spiraling along the cochlea.

**FIGURE 4:**
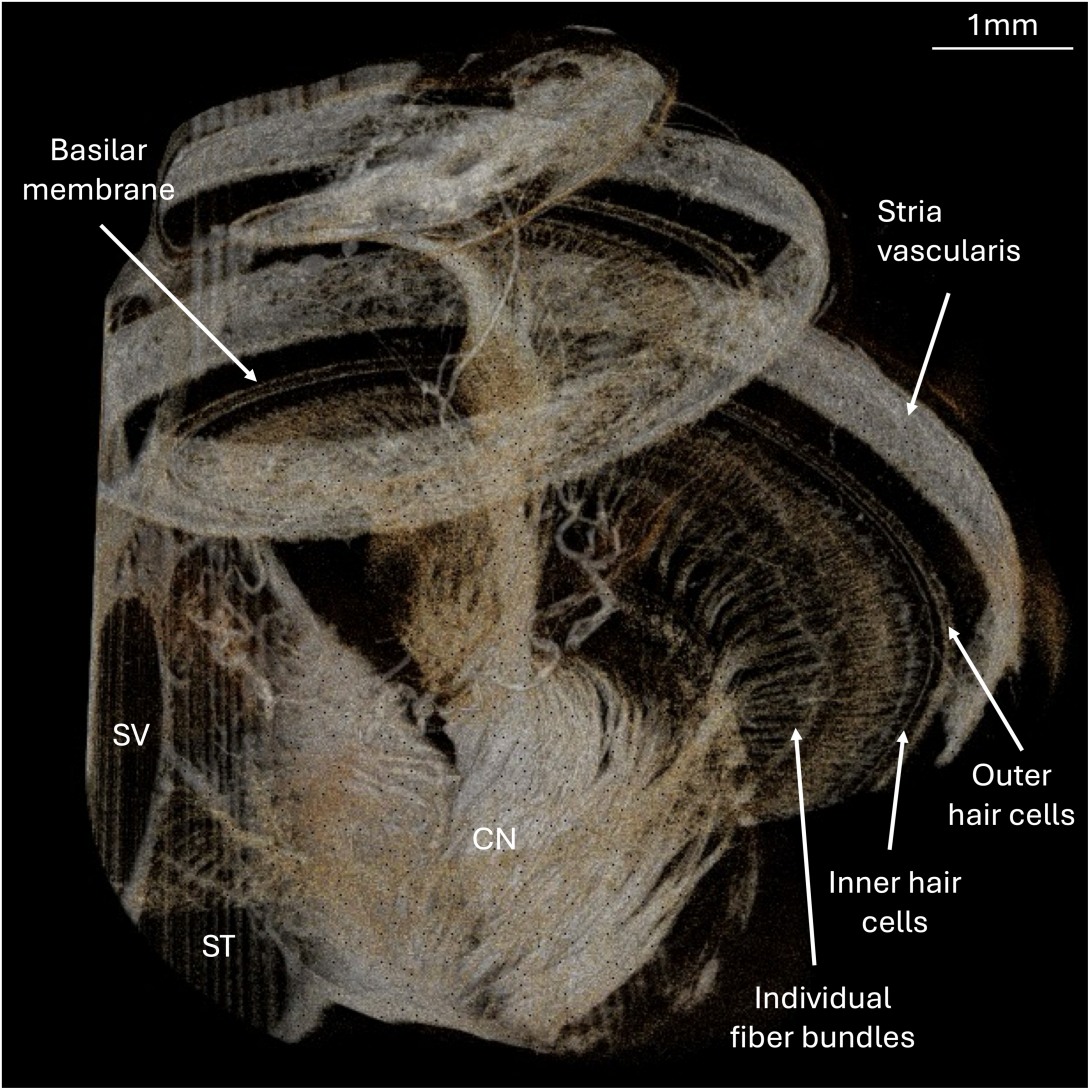
Cochlear XRM of human temporal bones at 4x magnification reveals a mosaic of neatly arranged rows of sensory and non-sensory cells in the organ of Corti. 3D reconstruction of XRM images enables distinct delineation of individual nerve fiber bundles, rows of inner and outer hair cells, and the stria vascularis. Scale bar is 1 mm.

**FIGURE 5:**
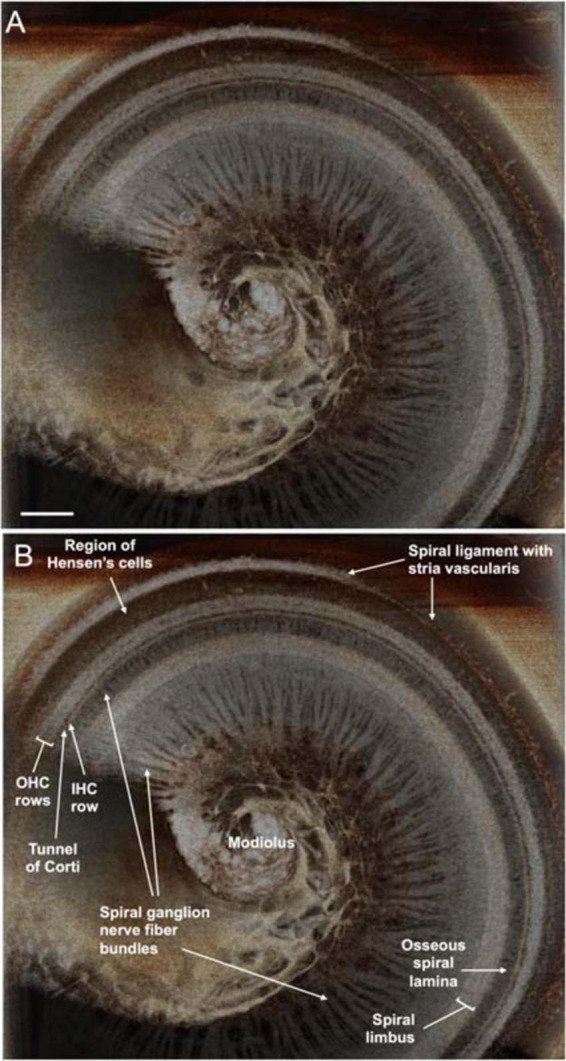
Digital reconstruction of the *en face* view of the organ of Corti generates a virtual whole mount preparation. **(A)** A virtual *en face* view of the cochlea was generated from reconstructed grayscale XRM data by orienting the 3D volume parallel to the basal turn, creating a whole-mount-like visualization of the organ of Corti. A density-based color filter was applied during volume rendering to enhance visualization of anatomical boundaries. Distinct structures can be appreciated such as nerve fiber bundles within the modiolus, spiral limbus, individual nerve fiber bundles within the osseous spiral lamina, a single row of inner hair cells, the tunnel of Corti, three rows of outer hair cells, Hensen’s cells, and the stria vascularis with the spiral ligament. The porous core of the modiolus is in the center of the image. Scale bar is 1 mm. **(B)** An annotated version of **(A)** identifying the microanatomical features of the human organ of Corti.

Focused imaging with higher 4x magnification and 3D modeling of contrast-enhanced soft tissue of the intact cochlea was performed (Figure 4). Individual nerve fiber bundles, inner and outer hair cells, and the stria vascularis were visualized. A zoomed-in 3D view of the structures of the intact cochlea, including individual spiral ganglion nerve fiber bundles from the organ of Corti centralizing medially at the modiolus, can be observed in a fly-through animation within the scala vestibuli (Supplementary Video 2). Sectioning the 3D reconstruction of the cochlea *en face* (parallel) to the basal turn generated a virtual whole mount (Figure 5, refer to Figure 4a in Landegger et al., 2019 for an example of a dissected cochlear whole-mount preparation and Supplementary Figure 1 for schematic representation). In addition to the tunnel of Corti, distinct cellular structures of the cochlea could be distinguished, including nerve fiber bundles within the modiolus and osseous spiral lamina, a single row of inner hair cells, clustered rows of the outer hair cells, Hensen’s cells, and stria vascularis with the spiral ligament.

**FIGURE 6:**
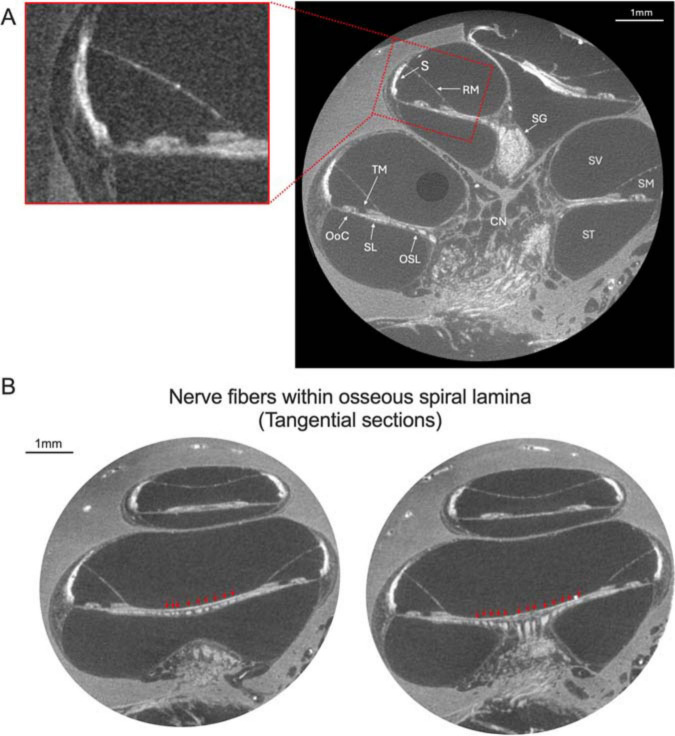
XRM affords high resolution of cochlear microanatomy. **(A)** A mid-modiolar section at 4x magnification of the scala media revealing organ of Corti (OoC), osseous spiral lamina (OSL), Reissner’s membrane (RM), spiral limbus (SL), and stria vascularis (S). Individual spiral ganglion neuron (SG) somata can be seen in the mid-modiolar section. The black circle in the scala vestibuli lumen is a ring artifact related to slight misalignment between the image reconstruction axis and the true cochlear mid-modiolar axis during imaging. **(B)** A tangential section from the mid-modiolus showed contrast-enhanced radial SG fiber bundles within the OSL (red arrows) that converge medially toward the cochlear nerve (CN). SV, scala vestibuli; SM, scala media; ST, scala tympani.

To assess the mid-modiolus view, which is most commonly used in otopathology, 2D orthogonal cross-sections of the cochlea were obtained (Figure 6). Many micro-anatomical structures can be observed in high resolution with individual structure delineation, such as the organ of Corti, tunnel of Corti, tectorial membrane, and cross-sectional spiral ganglion nerve fiber bundles in the osseous spiral lamina (Figure 6A). A tangential section from the mid-modiolus showed spiral ganglion nerve fiber bundles within the osseous spiral lamina that merge medially into the modiolus to form the cochlea nerve (Figure 6B).

To evaluate the degree of resolvability of the cells in the organ of Corti, we generated a reference table of the voxel sampling needed for the inner and outer hair cells based on well-established dimensions of these cells in humans (Buswinka et al., 2024; Giese et al., 2024a; Goutman et al., 2015; Lim and Brichta, 2016; Table 1). This practically translates to the object appearing as a diffuse density (blurry blob) when 1–2x the voxel size, individual structures become identifiable when 3–4x the voxel size, and internal structures are clearly delineated when 8–10x the voxel size. Based on human inner and outer hair cell diameters of 8–10 μm and 6–8 μm, respectively, and the minimal sampling needed for each degree of structural resolvability, minimum voxel sizes of 3.00 and 2.33 μm are needed for delineation of individual cells. At 4x magnification with XRM, the lower IQR was 4.10 μm voxel, resulting in voxel sampling of 1.73x and 1.35x for inner and outer hair cells, respectively, smaller than the minimum voxels needed to distinctively resolve each cell type. Despite the voxel sampling limitations, contrast-enhanced tissues were detected with distinct structural borders.

**TABLE 1:** The degree of structural resolvability of the inner and outer hair cells by XRM is dependent on the size of the target area of interest and the number of voxel sampling per area.

Structural resolvability	Example in organ of Corti	Voxel sampling needed^a^	Voxel size (μm)	
			Inner hair cell (8–10 μm)	Outer hair cell (6–8 μm)
Not reliably detectable	Expected structure not visualized	<2	–	–
Feature detectability	Blurry blob	2	4.50	3.50
Morphological delineation	Discrimination of external boundaries of cells	3–4	2.25–3.00	1.75–2.33
Substructural resolution	Resolving internal organization such as organelles	8–10	0.90–1.13	0.70–0.88

*^a^*Sample values are on a continuous scale, values in between categories are intermediate sample regime.

### 3.4 Case study quantifying biometrics of the human cochlea with XRM

Using 2D XRM orthogonal and H&E stained mid-modiolus sections (one human specimen each), we measured the biometric differences in the organ of Corti from base to apex and correlated this with its tonotopic map (Greenwood, 1990; Sridhar et al., 2006; Stakhovskaya et al., 2007). Radiological characterization of the human inner ear remains limited, particularly for quantitative biometric measurements of cochlear morphology. Accordingly, the width-to-height ratio observed with XRM was compared to previously reported measurements in mice (Ehret and Frankenreiter, 1977; Soons et al., 2015). We observed an increase in width-to-height ratio of the human organ of Corti from the basal to higher turns in both XRM and H&E-stained histology sections, ranging from 2.1 to 2.6 and 2.4 to 2.9, respectively (Table 2). This increasing trend is consistent with previously reported width-to-height ratios in mice obtained by two-photon microscopy, which ranged from 1.8 to 3.9 (Ehret and Frankenreiter, 1977; Soons et al., 2015). We observed that, compared to the rate of change of the width-to-height ratio in mice (52.5%), the human organ of Corti had a lower rate of change corresponding to 13.5% using XRM and 17.0% using histology, which may be due to species-specific differences in anatomy and auditory thresholds. The human H&E specimen had mild deterioration, likely related to processing artifacts during embedding and sectioning, with the apex being sectioned slightly off-center from mid-modiolus.

**TABLE 2:** Case study measurements of the width-to-height ratio of the human organ of Corti using XRM.

Location of the organ of Corti	Distance from round window	Characteristic frequency range (kHz)^a^	Width-to-height ratio		
			XRM in human	Histology in human	In mice^b^
Basal lower	0–10%	20.0–12.0	2.1	2.4	1.8
Basal upper	10–30%	12.0–5.0	2.1	2.4	–
Middle lower	30–45%	5.0–2.8	2.4	2.6	2.6
Middle upper	45–70%	2.8–1.0	2.5	2.9	–
Apex	70–100%	1.0–0.2	2.6	–	3.9

The cochlear length in the mid-modiolar plane follows similar trends in XRM images and histological sections, increasing from the basal to the apical turn. Human specimens were matched on age and post-mortem interval.

^a^Sources for the characteristic frequency ranges were: Greenwood, 1990; Sridhar et al., 2006; Stakhovskaya et al., 2007.

^b^Sources of the width-to-height ratios in mouse cochlea were: Ehret and Frankenreiter, 1977; Soons et al., 2015.

## 4 Discussion

Here, we establish a sectioning-free otopathology pipeline for fixed human inner ear specimens using contrast-enhanced XRM, enabling *ex vivo* visualization of cochlear microanatomy while remaining within the trimmed temporal bone. XRM generated high-resolution 2D and 3D views of cochlear and vestibular structures and supported proof-of-concept biometric analysis of the organ of Corti. Quantitative morphometric measurements of the human organ of Corti have been typically reported as individual cell dimensions using 2D histological methods rather than geometric ratios, and organ-level width-to-height relationships have not been systematically characterized within the intact cochlea. Digital sectioning of the cochlea *ex vivo* within the temporal bone typically requires sub-micron resolution modalities such as synchrotron imaging, although such studies have focused primarily on tonotopic mapping and gross inner ear quantitative analysis, with comparatively limited analysis of organ of Corti microanatomy (Giese et al., 2024a; Li et al., 2021; Li et al., 2025).

XRM can image objects of various sizes, with smaller FOV generally permitting higher nominal resolution. In this study, our goal was to capture the entire cochlea, rather than sub-sections of the lumen, to reconstruct the cochlea in 3D and assess gross anatomical variation from the base to apex within a single dataset. This approach is particularly relevant because some cochlear insults, including noise exposure and presbycusis, can exhibit region-specific pathology corresponding to frequency-specific hearing loss (Nelson and Hinojosa, 2006; Wu et al., 2021). Traditionally, capturing a large area of interest would require the object to be placed farther from the X-ray source, which can increase beam divergence and reduce photon collection efficiency, ultimately lowering spatial resolution. A key advantage of XRM is that high resolution can be maintained even at lower geometric magnification because secondary optical magnification compensates for the increased source-sample distance. Accordingly, we used lower magnification for imaging the cochlea and vestibular system together (average FOV 17.0 mm; Curtis et al., 2023; Geneci et al., 2022; Krombach et al., 2005; Spedaliere et al., 2025; Teixido et al., 2015) and higher magnification for imaging the cochlea alone (average FOV 9.2 mm; Curtis et al., 2023; Meng et al., 2016; Shin et al., 2013; Singla et al., 2015).

However, larger FOVs require longer exposure times per projection because X-rays must traverse greater tissue thickness, increasing total scan duration. In our study, scan time reached 13.3 h at 4.0x magnification and was determined by both exposure time and the number of projections acquired. To reduce exposure time, iodine staining protocols can be optimized to increase soft tissue enhancement and, by adjusting specimen orientation, to minimize its radius perpendicular to the source beam. When determining specimen orientation, we considered the dimensions of the human cochlea: the transverse diameter (A) averages 9.2 mm (range 8.4–10.4 mm; Curtis et al., 2023; Erixon and Rask-Andersen, 2013; Ritman, 2007) at the basal turn, whereas the perpendicular diameter (B) averages 6.5 mm (range 5.7–6.9 mm; Meng et al., 2016; Ritman, 2007). Although the B dimension is smaller, the A axis was aligned parallel to the source beam to maintain an even path along the beam during rotational imaging despite asymmetry along the cochlear spiral (base to apex). Potential reconstruction artifacts arising from this geometry can be minimized by using a physical metal filter during acquisition and beam hardening correction during the 3D reconstruction. Scan duration can also be reduced by decreasing the angular sampling frequency (projections). While this does not change the spatial resolution, which is dependent on the optics, it reduces the fidelity of 3D reconstruction. Our protocol trimmed the temporal bone plug to the otic capsule to reduce attenuation from surrounding bone and improve cochlear soft-tissue contrast. This requirement reflects the trade-off between specimen size, FOV, and resolution: XRM favors smaller specimens positioned close to the source, whereas the parallel-beam geometry and high photon flux of SR-PCI permit a larger FOV at comparable resolution. Thus, SR-PCI is advantageous for larger temporal bone preparations, while contrast-enhanced XRM provides a more accessible laboratory-based approach optimized for trimmed *ex vivo* specimens. Although decalcification was not required in our workflow, it may further improve XRM resolution and soft-tissue contrast by reducing attenuation from residual mineralized bone.

Another parameter to consider when imaging biological specimens is the radiation received by the tissue. Although XRM operates at keV energies and does not induce radioactivity (Gianoncelli et al., 2015; Shinohara and Ito, 1991), prolonged exposure can generate ionization events that disrupt chemical bonds and contribute to microscopic tissue degeneration (Brenner and Hall, 2007; Hirshfeld et al., 2018). Therefore, “non-destructive” in this study refers to preservation of the fixed specimen without physical sectioning, decalcification, or consumption during imaging, rather than the absence of radiation effects. When imaging a specific cochlear sub-region (e.g., the tonotopic region corresponding to 4,000 kHz), the X-ray beam necessarily traverses all tissue in its path, with unintentional radiation exposure to non-target tissue. This may limit repeat imaging of the same specimen if long exposure times are required. Although the effect on structural integrity is typically minimal, it is recommended to first image larger regions and then perform repeat imaging of smaller regions to reduce non-uniform radiation exposure on the global scan. Notably, the iodine-based contrast used here offers the advantage of reversible tissue binding (Bommakanti et al., 2022), allowing XRM-imaged specimens to remain available for subsequent validation assays. Therefore, downstream histology or immunohistochemistry can be used to assess imaging-related structural changes versus the contralateral non-imaged ear, although mild tissue shrinkage and other potential artifacts introduced during specimen processing should be considered when interpreting the results.

3D reconstruction of the human cochlea using XRM images showed clear delineation of the single row of inner hair cells separated by the tunnel of Corti, with a thicker row of the outer hair cells along the cochlear spiral. This level of cellular resolution is consistent with the size of the cells and XRM’s resolution capabilities. The ability to resolve cellular structures depends on the relationship between voxel size and target structure size (a voxel is the 3D analog of a pixel and represents the smallest sampled volume within the reconstructed dataset). According to the Nyquist-Shannon Sampling Theorem dictating the minimum sample frequency required to avoid signal aliasing (Shannon, 1949), at least two samples per the smallest resolvable period are needed to detect the object (Table 1). We used 4x magnification to capture the entire cochlea within the FOV while maintaining sufficient resolution of gross microanatomical structures. Voxel sampling remained below two voxels across the dimensions of both inner and outer hair cells, precluding delineation of individual cell boundaries. However, the use of iodine further enhanced the soft tissue of the cochlea to increase the signal-to-noise ratio, which enabled visualization of the external boundaries of the cells, especially of inner hair cells that are larger than outer hair cells. Detailed morphometric analyses of hair-cell arrangement have generally required higher-resolution methods, including SEM, high-resolution light microscopy, or histology (Girdlestone et al., 2020). Single-cell resolvability is lower compared to SR-PCI with the same FOV, but the difference is small considering the significant reduction in the beam source used in regard to coherence and intensity of the X-rays. Although sub-micrometer imaging has been achieved using XRM by decreasing the FOV to 1–2 mm (Bidola et al., 2017), XRM is primarily used to achieve resolution in the micrometer scale. Future studies optimizing targeted XRM acquisition of smaller regions may improve cellular delineation and enable selected cell-row or regional morphometric measurements.

Additionally, we performed a case study to assess the feasibility of XRM for biometric analysis of the organ of Corti. The width and height increase at different rates from base to apex: the width expands more rapidly due to the geometric and mechanical characteristics of the cochlea. While the luminal diameter of the cochlear duct decreases from base to apex, the basilar membrane widens to accommodate low-frequency sound processing (Liu et al., 2015; Tani et al., 2021). In contrast, increase in organ of Corti height is mechanically constrained by the cytoarchitecture of supporting cells (e.g., Deiters’ and pillar cells), which contribute to the flexibility and stiffness ratio along the cochlea (Giese et al., 2024a; Liu et al., 2015; Soons et al., 2015). Consequently, the width-to-height ratio increases from the base to the apex, as reported in prior studies in humans and mice using modalities where cellular and subcellular delineation is possible (e.g., SEM and two-photon microscopy) (Giese et al., 2024a; Glueckert et al., 2005a; Glueckert et al., 2005b; Kawano et al., 1996; Soons et al., 2015). As a proof of concept, we compared mid-modiolar views of the human organ of Corti obtained by XRM with H&E histological sections (the reference standard for human otopathology) and observed an increasing width-to-height ratio from base to apex as hypothesized. Because quantitative human organ of Corti datasets remain limited, we quantified the width-to-height ratio in mouse organ of Corti, which showed a similar base-to-apex increase but at a steeper rate (∼1.5x compared to the human specimen). This difference likely reflects species-specific differences in cochlear morphology, as mice have fewer cochlear turns (1.7 vs. 2.5 in humans) mapping across a broader frequency range than humans.

Among radiological imaging methods capable of resolving the entire cochlea, SR-PCI has the highest potential to fully resolve individual cells of the organ of Corti, although it has not yet demonstrated equal spatial resolution of histology with high magnification. Future validation studies should therefore include larger numbers of matched specimens evaluated by XRM, SR-PCI, and histology. However, the novelty of our study is not that XRM exceeds other *ex vivo* methods like optical or electron microscopy for single-cell morphometry, or SR-PCI in terms of spatial resolution, but rather that it extends biometric analysis to an accessible sectioning-free, radiological workflow that preserves the native architecture of the human cochlea in 3D within the temporal bone. Beyond binary assessment of whether the organ of Corti cellular regions are present or absent along the cochlear spiral, which may indicate hair cell loss within a given tonotopic region, quantitative morphometric measurements could provide additional insight when correlated with clinical audiometry. Ultimately, a reference library of high-fidelity *ex vivo* radiological otopathology datasets with biometric measurements, paired with donor clinical histories and audiometric data when available, could facilitate correlations between cochlear microanatomy and hearing phenotypes, such as altered width-to height ratios following acoustic trauma. In addition, because the basilar membrane is not consistently distinguishable in many radiological imaging modalities, including XRM, comparative studies with histological datasets where the basilar membrane is clearly resolved will be valuable to assess whether combined regions of interest can serve as biologically valid proxies for individual substructures.

The results of this study should be interpreted in the light of several limitations, some of which are common to otopathology studies using rare human tissue. First, the case study used one human cochlea per method (XRM and H&E) due to the limited specimens available from post-mortem donations. Second, the SR-PCI image included in this study was derived from previously published legacy data (Iyer et al., 2018) and was used only as a qualitative contextual comparator. Because SR-PCI acquisition, detector technology, phase retrieval, reconstruction, and image-processing methods have advanced since that dataset was acquired (Elfarnawany et al., 2017; Giese et al., 2024b; Li et al., 2025; Rohani et al., 2019; Rohani et al., 2020), this comparison should not be interpreted as a benchmark of contemporary SR-PCI performance relative to XRM. Contemporary SR-PCI remains a higher-resolution radiological approach than XRM and would be an important comparator in future quantitative and larger scale validation studies using matched specimens imaged with optimized XRM, SR-PCI, and histological workflows. Third, given the stochastic nature of post-mortem donations, the samples in this study were obtained from patients with varying demographic and clinical backgrounds, and procured at variable PMI, which may introduce heterogeneity. Fourth, human organ of Corti biometrics assessed via XRM were compared with previously reported measurements from mouse due to the lack of comparable human data in the literature. Finally, a moderate computation load is required for processing XRM images and a workstation-class computer is needed to perform analyses. Future studies incorporating larger cohorts of human data, including audiometric thresholds, are warranted to correlate individuals’ audiological profiles with cochlear pathology observed with XRM.

In conclusion, XRM enables high-resolution, non-destructive *ex vivo* imaging of fixed human inner ear specimens retained within trimmed temporal bone, with greater accessibility and lower cost than synchrotron-based systems. Our method using a reversible contrast stain may preserve prepared specimens for downstream studies such as immunohistochemistry and spatial proteomics. While XRM enables quantitative biometric analysis of the microanatomical structures of the human organ of Corti, further large-scale analyses are warranted to elucidate the relationship between anatomical measurements and otopathology, clinical etiologies, and audiological profiles.

## Data Availability

The original contributions presented in the study are included in the article/Supplementary material, further inquiries can be directed to the corresponding authors.
