# Supplementary material for: High resolution, contrast-enhanced X-ray microscopy of the ex vivo human cochlea: technical feasibility and biometric case analysis
Source: Front Neurosci. 2026 Jul 15;20:1838204. doi: 10.3389/fnins.2026.1838204 (PMC13417989; doi:10.3389/fnins.2026.1838204)
Supplement: Supplementary file 1 [file Data_Sheet_1.pdf]

SUPPLEMENTAL MATERIAL *for* "High resolution, contrast-enhanced X-ray microscopy of the *ex vivo* human cochlea: Technical feasibility and biometric case analysis"

Video 1. Anatomy of the internal auditory canal and inner ear with segmentation of the contrast-enhanced nerve tissue and hair cells.

Video 2. Flythrough view within the lumen of the cochlea.

Supplemental Table 1. Characteristics of temporal bone specimen donors.

Specimen processing	Age	Sex	Cochlea laterality	Post-mortem interval, h
XRM	77	Male	R	19
XRM	77	Male	L	19
XRM[a]	72	Female	L	36
H&E[a]	73	Female	L	21

Caption: Specimens were processed for use in XRM or celloidin-embedded H&E staining experiments. Note: [a] Specimens were used for the biometric case study. Abbreviations: H&E, hematoxylin and eosin; XRM, X-ray microscopy.

Supplemental Figure 1. An annotated schematic of whole mount cochlear microanatomy.

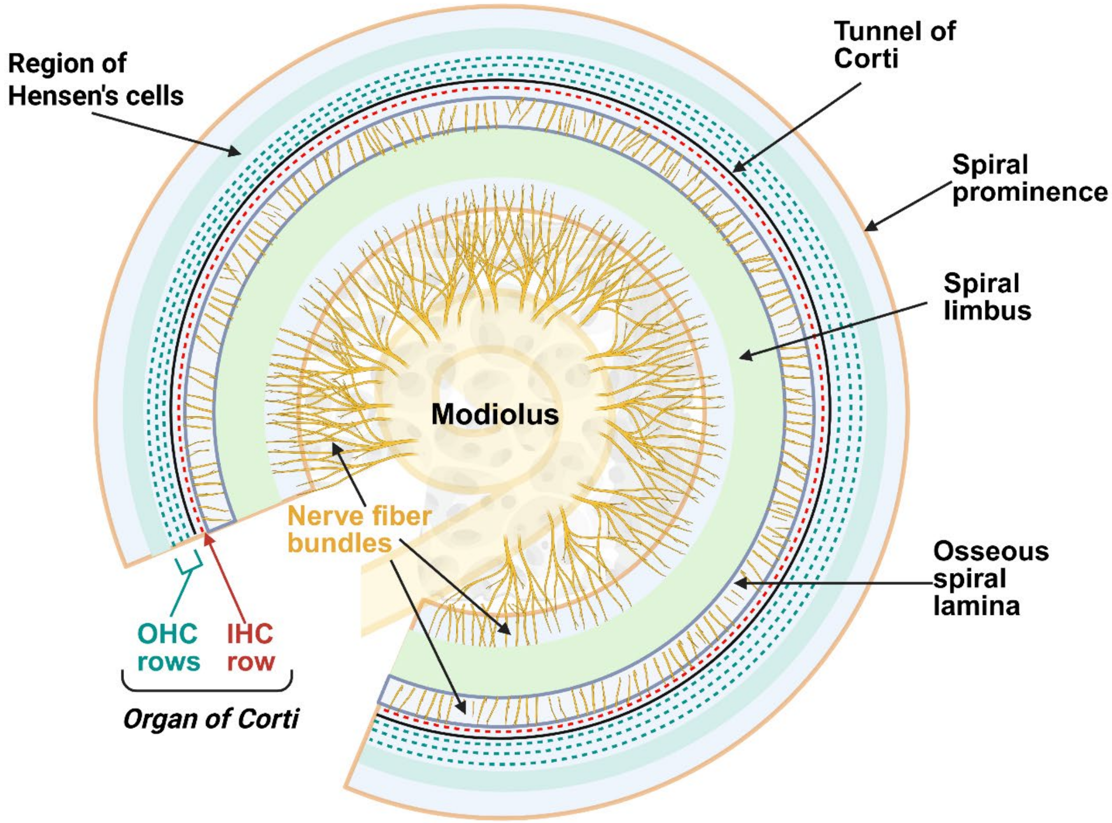

Abbreviations: IHC, inner hair cell; OHC, outer hair cell.
